# Interleukin-6 as a novel biomarker for the diagnosis of acute decompensated heart failure: a retrospective study

**DOI:** 10.3389/fcvm.2026.1800571

**Published:** 2026-03-24

**Authors:** Qiao-Mei Wu, Li-Juan Liu, Teng-Fei Li, Hai-Wei Deng, Jie Li, Yuan-Sheng Zhai, Xun Hu

**Affiliations:** 1 Department of Anesthesiology, Hospital of Stomatology, Guanghua School of Stomatology, Sun Yat-Sen University, Guangzhou, China; 2 Department of Cardiology, the First Affiliated Hospital, Sun Yat-Sen University, Guangzhou, China; 3 Key Laboratory on Assisted Circulation, Ministry of Health, Guangzhou, China

**Keywords:** acute decompensated heart failure, biomarker, diagnosis, inflammation, interleukin-6

## Abstract

**Background:**

Interleukin-6 (IL-6) is associated with the pathogenesis of heart failure (HF) and an increased HF risk in people without previous myocardial infarction. However, little is currently known about the correlation between IL-6 and acute decompensated heart failure (ADHF).

**Objective:**

To determine whether IL-6 could serve as a diagnostic biomarker for ADHF in Chinese patients without acute myocardial infarction.

**Patients and methods:**

A total of 122 patients were included in our study [58 ADHF, 64 chronic HF (CHF)]. Plasma IL-6 and N-terminal pro-B-type natriuretic peptide (NT-proBNP) were measured. Logistic regression analyses and a receiver operating characteristic (ROC) curve were performed to determine the diagnostic value of IL-6.

**Results:**

Plasma levels of IL-6 and NT-proBNP were higher in ADHF patients than CHF patients. IL-6 correlated positively with NT-proBNP, heart rate, and left ventricular end-diastolic dimension, and inversely with left ventricular ejection fraction. After adjustment, IL-6 remained independently associated with ADHF (OR = 1.461, 95% CI: 1.053–2.028, *p* = 0.023). The optimal cut-off value of IL-6 for ADHF diagnosis was 5.00 pg/mL, and the sensitivity, specificity, and Youden index were 81.0%, 87.5%, and 0.685, respectively.

**Conclusions:**

IL-6 is independently associated with ADHF and shows promising diagnostic accuracy. The cut-off of 5.00 pg/mL provides a novel reference for Chinese ADHF patients without AMI, offering clinical evidence for research on inflammation-related biomarkers of cardiovascular diseases. Combined use with NT-proBNP may enhance diagnostic accuracy, but prospective validation is needed.

## Introduction

Heart failure (HF), which results from various functional or structural heart disorders, represents a major public health burden worldwide whose prognosis is comparable to that of cancer. Currently, over 4,500,000 Chinese individuals have HF. With the growing incidence of HF risk factors (e.g., age, obesity, and hypertension), HF-related morbidity would likely increase significantly in the future. Acute decompensated heart failure (ADHF) is the leading cause of hospital admission among individuals age > 65 years (1), and more than one-third of the patients hospitalized because of ADHF are re-hospitalized or die within 90 days after the initial discharge (2).

Early intervention for ADHF has shown obvious improvement in the prognosis. Thus, a precise and prompt ADHF diagnosis is of great importance. A rapid diagnosis of ADHF, especially in the emergency department, has traditionally relied on a history of cardiovascular disorders and clinical manifestations, such as hypertension, deteriorating dyspnea, severe oedema, and rales. The approach was extremely dependent on the physicians’ experience, thereby resulting in misdiagnosis and missed diagnosis. N-terminal pro-B-type natriuretic peptide (NT-proBNP) is a well-established biomarker for the diagnosis of ADHF, and when combined with clinical presentation, it offers advantages over clinical assessment alone in evaluating symptom severity and predicting prognosis. Hence, NT-proBNP has become a critical parameter in the diagnostic algorithm of ADHF. However, despite the increased sensitivity and specificity of NT-proBNP, some patients such as those with extremely severe HF still experience misdiagnosis or missed diagnosis. Thus, identification of novel ADHF biomarkers to improve diagnostic accuracy is needed.

Since 1990, a large number of studies have demonstrated that inflammation is involved in the pathogenesis of HF (3, 4). Interleukin-6 (IL-6), which is an inflammatory cytokine, was once considered to originate exclusively from the immune system and to be involved in the hepatic production of downstream C-reactive protein. Nonetheless, it was recently validated that nearly all nucleated cell types residing in the myocardium, including cardiac myocytes, can secrete IL-6 in response to infection, tumor necrosis factor-α (TNF-α), and HF, thereby indicating the potential role of IL-6 in the development of HF. Subsequently, several clinical and experimental studies have gradually established the relationship between IL-6 and various cardiovascular disorders, such as left ventricular hypertrophy, coronary heart disease, and HF (5–8). However, controversies about IL-6's correlation with ADHF exist. Some reports showed that circulating IL-6 levels are markedly elevated in ADHF and thus could be an independent predictor of increased mortality (9). In contrast, a few researchers have found that IL-6 is only slightly increased in ADHF without change over time and lacks clinical usefulness in the long-term follow-up (10). Given this discrepancy, further investigation into the role of IL-6 in ADHF is warranted. In this study, we aimed to evaluate the diagnostic value of IL-6 in ADHF.

Moreover, plasma IL-6 levels are known to be markedly elevated in patients with acute myocardial infarction (AMI), where they reflect the inflammatory response to myocardial necrosis and predict adverse outcomes (9). In the context of diagnosing ADHF, including patients with AMI could introduce substantial confounding, as elevated IL-6 in such patients might be attributable to the acute ischemic event rather than to HF decompensation *per se*. This would obscure the specific relationship between IL-6 and ADHF and potentially reduce the diagnostic accuracy of IL-6 for ADHF. To isolate the association between IL-6 and ADHF that is driven by non-ischemic decompensation, we focused our investigation on patients without AMI. This focus is further supported by epidemiological evidence, such as that from the Framingham Heart Study, which indicates that IL-6 is associated with an increased risk of HF even in individuals without a history of myocardial infarction (11). Hence, in this study, we aimed to evaluate the association between IL-6 and ADHF and to assess its potential diagnostic value in Chinese patients without AMI using logistic regression analysis and receiver operating characteristic (ROC) curve.

## Patients and methods

### Study design and participants

**Figure 1:**
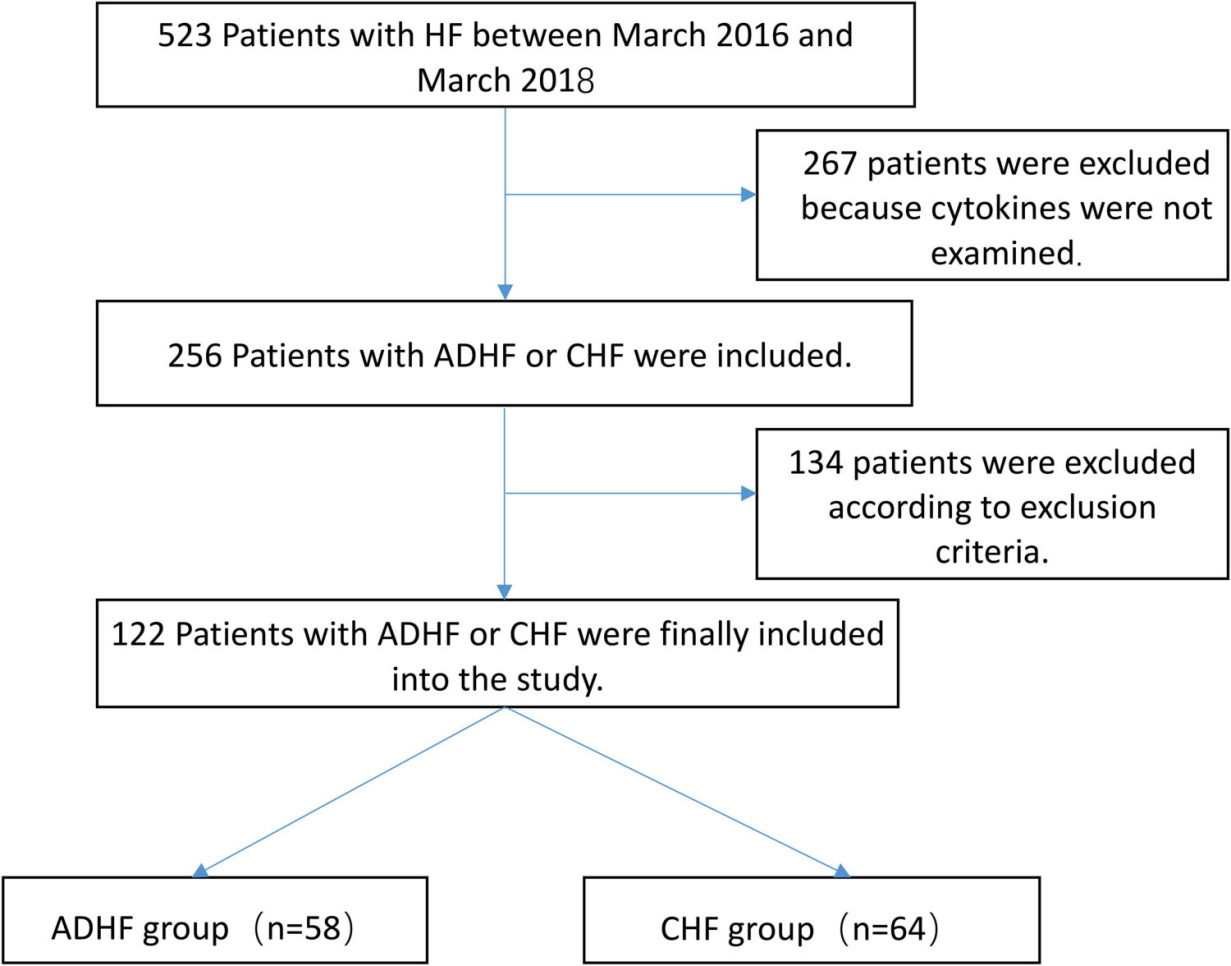
Study protocol. Exclusion criteria included the following: infection, acute myocardial infarction, moderate to severe renal failure (estimated glomerular filtration rate < 60 mL/min per 1.73 m^2^), malignancies, autoimmune disorders, treatment history of steroids and immunosuppressive drugs, rheumatoid disease, advanced liver disease, and myocarditis. HF, heart failure; ADHF, acute decompensated heart failure; CHF, chronic heart failure.

We reviewed patient data from the First Affiliated Hospital of Sun Yat-Sen University. A total of 523 consecutive patients with HF were admitted to our hospital between March 2016 and March 2018. We excluded 267 patients with HF whose inflammatory cytokines were not examined. Finally, this study included 58 ADHF inpatients without AMI with New York Heart Association (NYHA) functional class III or class IV upon admission to the hospital (Figure 1). The ADHF diagnosis was based on medical history, physical examination, electrocardiography, biomarkers, and results of chest x-ray and echocardiography in accordance with the guidelines of the American Heart Association and European Society of Cardiology (ESC) (12, 13). The underlying cardiac diseases of ADHF included hypertensive emergency, various cardiomyopathies (dilated cardiomyopathy, hypertrophic cardiomyopathy, ischemic cardiomyopathy, etc.), and valvular heart disease. In addition, 64 inpatients with stable chronic heart failure (CHF) were included as the control group (NYHA functional class I or class II). The underlying cardiac diseases of the participants with CHF were hypertension, stable coronary heart disease, various cardiomyopathies (dilated cardiomyopathy, hypertrophic cardiomyopathy, etc.), and valvular heart disease.

Patients with diseases affecting plasma IL-6 levels were considered ineligible for the study. Exclusion criteria included the following: infection, AMI, moderate to severe renal failure (estimated glomerular filtration rate <60 mL/min per 1.73 m^2^), malignancies, autoimmune disorders, history of treatment with steroids and immunosuppressive drugs, rheumatoid disease, advanced liver disease, and myocarditis. Patients with moderate to severe renal failure were excluded because renal dysfunction is known to independently elevate both IL-6 and NT-proBNP levels, which could confound the association between these biomarkers and ADHF. This exclusion criterion was applied to ensure that observed differences between groups reflect cardiac rather than renal contributions to biomarker elevation. AMI was verified according to the ESC's fourth universal definition of myocardial infarction, which required a rise and/or fall of troponin I with at least one value above the 99th percentile upper reference limit (URL), symptoms consistent with AMI and ST-segment elevation or depression in electrocardiograms (14).

The study protocol complied with the 1975 Declaration of Helsinki and was approved by the ethics committee of the First Affiliated Hospital of Sun Yat-Sen University (Approval No.: IIT- 2025-726). Due to the retrospective design of the present study, written informed consent was not required.

### Data collection

The following patient data were collected: (1) Demographics: age and sex; (2) Comprehensive medical history, including dilated cardiomyopathy, hypertrophic cardiomyopathy, and hypertension; (3) Symptoms and physical signs: dyspnea, orthopnea, severe edema, and rales; (4) Laboratory results: white blood cell (WBC) count, NT-proBNP, procalcitonin, troponin I, IL-6, interleukin-8 (IL-8), and interleukin-1β (IL-1β); and (5) Pharmacological therapy at admission, including the use of statins, angiotensin-converting enzyme inhibitor (ACEI), *β*-blockers, and mineralocorticoid receptor antagonist (MRA).

### Biomarker assays

Peripheral venous blood samples for comprehensive biomarker profiling, including WBC count, NT-proBNP, troponin I, IL-6, IL-8, and IL-1β, were collected from all patients after a 12-hour overnight fast, typically on the morning following hospital admission. This standardized protocol was implemented to ensure consistency of biomarker measurements and to avoid the confounding effects of food intake and diurnal variation. All samples were analyzed following the manufacturer's instructions and the laboratory's standard operating procedures. Plasma levels of inflammatory factors (IL-6, IL-8, IL-1β) and NT-proBNP were measured using chemiluminescent immunoassay (Automated Immunoassay Analyzer, Siemens Healthcare Diagnostics Inc., Munich, Germany). Plasma levels of other biomarkers were analyzed using conventional methods. All laboratory analyses were performed by trained technicians who were blinded to all clinical information, including patient group assignment, medical history, and echocardiographic findings. Samples were processed in random order to minimize potential batch effects. The blinding procedure was maintained throughout the entire laboratory analysis phase to prevent any bias in biomarker measurements.

### Echocardiography

The patients underwent M-mode and 2D echocardiography using a GE Vivid E9 ultrasonography machine (GE Healthcare, Philadelphia, Pennsylvania, USA). Standard parameters, including left ventricular end-diastolic dimension (LVEDD) and left ventricular ejection fraction (LVEF), were measured by two experienced technicians who were blinded to group allocation. Disagreements were resolved through consultation with a third technician. LVEF was calculated from the apical four-chamber position by the area–length method.

### Statistical analysis

Continuous variables were expressed as mean ± standard deviation or median and interquartile range based on distributional normality, and categorical variables were presented as percentages. The Shapiro–Wilk test was performed to determine whether the variables were normally distributed. Comparison of continuous variables between two groups was performed using a two-sample Student's t-test or non-parametric test, depending on the distributional normality. Categorical variables were analyzed by chi-squared tests.

Correlation analyses were conducted to examine the relationships between IL-6 and other key variables. In the overall study population, we assessed the correlations between IL-6 and key clinical and laboratory parameters, including NT-proBNP, other inflammatory biomarkers (IL-8, IL-10), echocardiographic indices (LVEDD, LVEF), as well as blood pressure and heart rate (HR). Subsequently, to further explore the association between IL-6 and HF severity, a separate correlation analysis was performed within the CHF group, evaluating the relationship of IL-6 with NT-proBNP, NYHA functional class, and LVEF.

Binary logistic regression was performed to identify variables independently associated with ADHF and to calculate odds ratios (ORs) with 95% confidence intervals (CIs). Age and gender were included as core demographic confounders based on clinical and epidemiological rationale. NT-proBNP was incorporated as the reference standard biomarker for HF. Additional covariates were considered for inclusion based on: (1) established pathophysiological relevance to HF or inflammation (LVEF); (2) significance in univariate analysis at *p* < 0.05; and (3) avoidance of multicollinearity.

Multicollinearity among the candidate predictors was assessed by calculating the variance inflation factor (VIF) and condition index using linear regression. As the observed distributions of the studied biomarkers were skewed, data were subjected to logarithmic transformation before the analyses.

In addition, a sensitivity analysis was conducted to assess the influence of extreme IL-6 values. Patients with IL-6 levels above the 95th percentile of the entire study population were excluded, and multivariable logistic regression was repeated adjusting for the same set of candidate covariates considered in the primary analysis.

Finally, a ROC curve was employed to determine IL-6's sensitivity, specificity, Youden index, and cut-off value for ADHF diagnosis. A *p*-value <0.05 for two-tailed tests was considered statistically significant. The Statistical Package for Social Sciences (SPSS) version 26.0 (IBM Corporation, Armonk, New York, NY, USA) was used for all statistical analyses.

## Results

### Clinical characteristics of the study population

The clinical characteristics of the participants are shown in Table 1. A total of 122 inpatients were recruited in this study and were divided into the ADHF group (58 participants with NYHA functional class III and IV) and CHF group (64 patients with NYHA functional class I and II). No significant differences between the two groups were observed in baseline demographic data (age, sex) or pharmacological therapy use. The HR of the patients with ADHF was markedly higher than that of the patients with CHF, although the systolic blood pressure (SBP) and diastolic blood pressure (DBP) were comparable between the groups. The underlying cardiac diseases of HF mainly consisted of hypertensive heart disease, stable coronary heart disease, cardiomyopathy, and valvular heart disease, with no significant differences in distribution between the groups. Moreover, patients with ADHF had greater LVEDD and worse cardiac function (LVEF).

**Table 1:** Demographic and clinical characteristics of study participants.

Variables	ADHF group	CHF group	*p* value
	*n* = 58	*n* = 64	
Age (years)	64.0 ± 14	62.8 ± 13	0.638
Gender (%)			0.877
Male	30 (51.7)	34 (53.1)	-
Female	28 (48.3)	30 (46.9)	-
HR (bpm)	118.9 ± 17	74.0 ± 9.0	< 0.001
SBP (mmHg)	132 (118, 157)	132 (120, 165)	0.756
DBP (mmHg)	72 (65.5, 86.5)	75 (70, 90)	0.148
NYHA I (%)	0	31 (48.4)	-
NYHA II (%)	0	33 (51.6)	-
NYHA III (%)	28 (48.3)	0	-
NYHA IV (%)	30 (51.7)	0	-
Underlying cardiac diseases			0.504
Hypertension (%)	13 (22.4)	21 (32.8)	-
Coronary heart disease (%)	10 (17.2)	8 (12.6)	-
Cardiomyopathy (%)	22 (38)	19 (29.7)	-
Valvular heart disease (%)	13 (22.4)	16 (25.0)	-
Echocardiographic parameters			
LVEDD (mm)	57.5 ± 7.2	52.9 ± 5.9	< 0.001
LVEF (%)	45 (40, 55)	50 (46, 60)	< 0.001
Pharmacological therapy			
ACEI (%)	56 (96.5)	61 (95.3)	0.730
MRA (%)	55 (94.8)	62 (96.9)	0.569
*β*-blocker (%)	49 (84.5)	54 (84.4)	0.987
Statin (%)	15 (25.9)	15 (23.4)	0.756

ADHF, acute decompensated heart failure; CHF, chronic heart failure; HR, heart rate; SBP, systolic blood pressure; DBP, diastolic blood pressure; NYHA, New York Heart Association; LVEDD, left ventricular end-diastolic dimension; LVEF, left ventricular ejection fraction; ACEI, angiotensin-converting enzyme inhibitor; MRA, mineralocorticoid receptor antagonist. Data were expressed as mean ± standard deviation (SD) or median and interquartile range or *n* (%).

### Biomarkers

As shown in Table 2, no significant differences were observed in alanine aminotransferase (ALT) or serum creatinine, indicating preserved hepatic and renal function in most participants upon admission. Similarly, plasma levels of WBC, hemoglobin, thyroid-stimulating hormone (TSH), free triiodothyronine (FT3), free thyroxine (FT4), triglyceride (TG), total cholesterol (TC), and low-density lipoprotein cholesterol (LDL-C) also showed no significant differences between the groups. In contrast, plasma NT-proBNP levels were significantly elevated in ADHF patients compared to the CHF group. Furthermore, analysis of inflammatory factors revealed significantly higher plasma IL-6 levels in the ADHF group compared to the CHF group [13.00 (6.03, 31.85) pg/mL vs. 2.85 (1.10, 4.08) pg/mL, *p* < 0.001]. However, plasma levels of IL-8 and IL-10 did not differ significantly between the two groups (Table 2).

**Table 2:** Plasma biomarkers.

Variables	ADHF	CHF	*p* value
	*n* = 58	*n* = 64	
WBC (*10^9^/L)	6.83 ± 1.68	6.90 ± 1.45	0.820
Haemoglobin (g/L)	124.5 (111.5, 137.2)	126 (121.0, 132.0)	0.819
Creatinine (µmol/L)	79.7 ± 16.4	80.3 ± 13.3	0.823
ALT (U/L)	26.8 ± 10.8	27.4 ± 8.1	0.698
TSH (mIU/mL)	2.09 ± 0.64	2.00 ± 0.63	0.444
FT3 (pmol/L)	4.49 ± 0.64	4.53 ± 0.48	0.702
FT4 (pmol/L)	16.50 ± 2.16	16.50 ± 1.73	0.899
TC (mmol/L)	4.20 (3.60, 5.15)	4.50 (3.70, 5.08)	0.637
LDL-C (mmol/L)	2.53 (2.10, 3.29)	2.53 (2.16, 3.30)	0.778
TG (mmol/L)	1.52 ± 0.54	1.50 ± 0.43	0.903
NT-proBNP (pg/mL)	5,919.50 (3,849.75, 14,622.00)	243.00 (86.00, 588.50)	< 0.001
IL-10 (pg/mL)	5.00 (5.00, 5.00)	5.00 (5.00, 5.00)	0.385
IL-8 (pg/mL)	28.50 (16.38, 41.30)	27.10 (10.45, 47.55)	0.988
IL-6 (pg/mL)	13.00 (6.03, 31.85)	2.85 (1.10, 4.08)	< 0.001

ADHF, acute decompensated heart failure; CHF, chronic heart failure; WBC, white blood cell; ALT, alanine aminotransferase; TSH, thyroid stimulating hormone; FT3, free triiodothyronine; FT4, free thyroxine; TC, total cholesterol; LDL-C, low density lipoprotein-cholesterol; TG, triglyceride lipase; NT-proBNP, N-terminal pro–B-type natriuretic peptide; IL-10, interleukin-10; IL-8, interleukin-8; IL-6, interleukin-6.

### Correlation analyses

**Figure 2:**
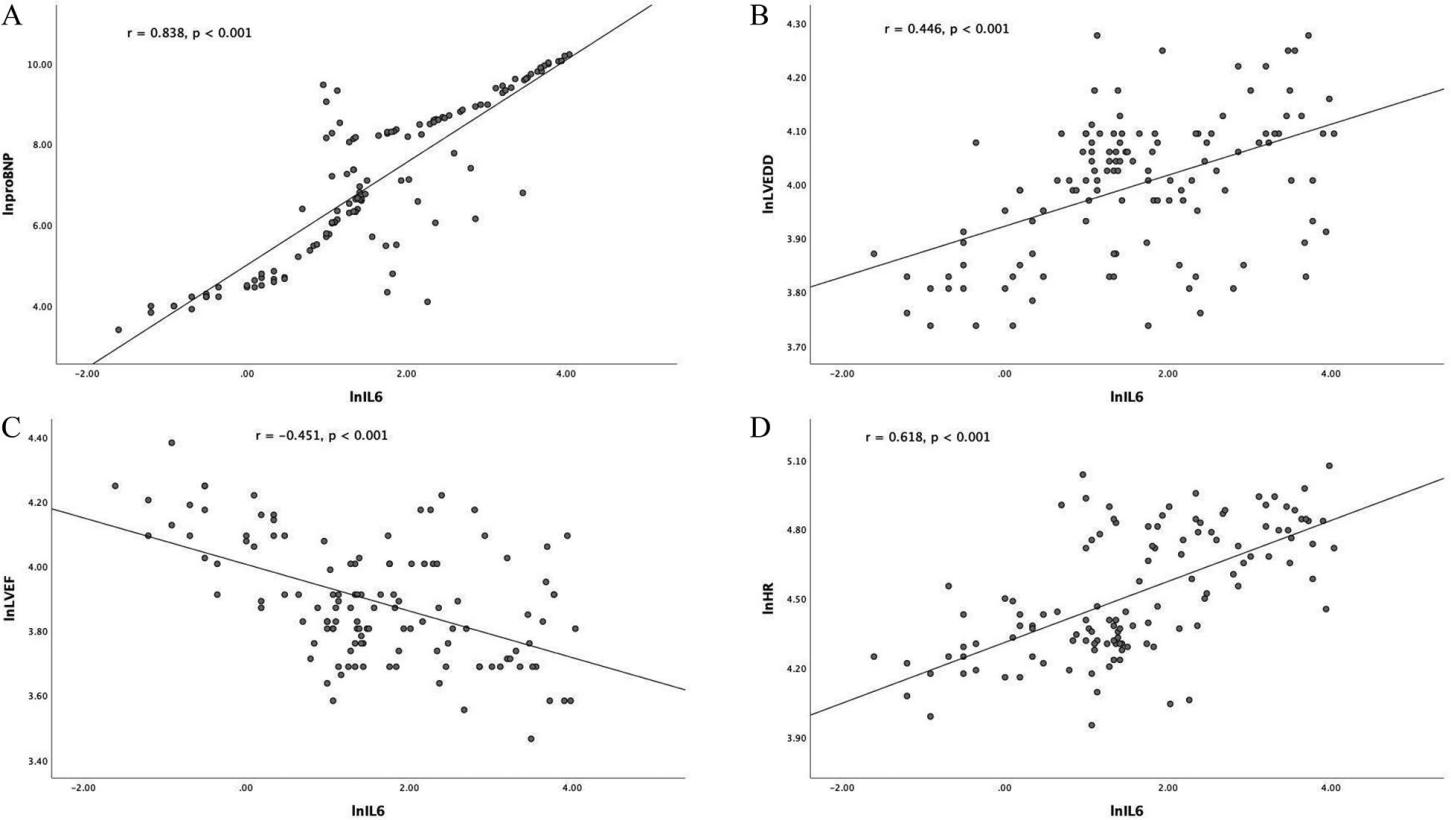
Correlation analyses. All biomarker and clinical variables were log–transformed to approximate normal distribution before analysis. Pearson correlation coefficients (r) and corresponding *p*-values are shown for each panel. After natural logarithmic transformation, IL-6 was positively associated with NT-proBNP (r = 0.838, *p* < 0.001) **(A)**, LVEDD (r = 0.446, *p* < 0.001) **(B)** and HR (r = 0.618, *p* < 0.001) **(D)**, and inversely associated with LVEF (r = −0.451, *p* < 0.001) **(C)**. NT-proBNP, N-terminal pro-B-type natriuretic peptide; IL-6, interleukin-6; LVEDD, left ventricular end-diastolic dimension; LVEF, left ventricular ejection fraction; HR, heart rate.

NT-proBNP is widely recognized as the diagnostic gold standard for ADHF. Consequently, we performed correlation analyses between NT-proBNP and IL-6, HR, LVEDD, and LVEF after natural logarithmic transformation. NT-proBNP exhibited significant positive correlations with IL-6 (r = 0.838, *p* < 0.001), HR (r = 0.736, *p* < 0.001), and LVEDD (r = 0.534, *p* < 0.001). A significant inverse correlation was observed with LVEF (r = −0.572, *p* < 0.001). Given the significant differences observed in IL-6, HR, LVEDD, and LVEF between groups, we further analyzed the relationships between IL-6 and these parameters. Specifically, IL-6 showed positive correlations with HR (r = 0.618, *p* < 0.001) and LVEDD (r = 0.446, *p* < 0.001) and a negative correlation with LVEF (r = −0.451, *p* < 0.001) (Figure 2).

Given the significant difference in IL-6 levels between ADHF and CHF groups, we further investigated the relationship between IL-6 and disease severity within the CHF control group (*n* = 64). Among these clinically stable patients, plasma IL-6 levels showed strong positive correlations with NT-proBNP (r = 0.738, *p* < 0.001) and NYHA functional class (r = 0.607, *p* < 0.001). A significant negative correlation was observed between IL-6 and LVEF (r = −0.523, *p* < 0.001). These findings indicate that even among stable CHF patients, higher IL-6 levels are associated with more advanced HF.

### Logistic regression model

To identify factors independently associated with ADHF, binary logistic regression was performed including age, gender, NT-proBNP, IL-6, and LVEF as candidate predictors. In this multivariable model, IL-6 remained significantly associated with ADHF (OR = 1.461, 95% CI: 1.053–2.028, *p* = 0.023), as did NT-proBNP (OR = 1.003, 95% CI: 1.001–1.006, *p* = 0.011).

Collinearity diagnostics revealed moderate multicollinearity between IL-6 and NT-proBNP (VIF values of 7.099 and 7.302, respectively), consistent with their known biological correlation. All other variables had VIF values below 1.2. The condition index was 21.6, indicating no severe overall collinearity. Given the theoretical importance of both biomarkers and their distinct pathophysiological roles, both were retained in the model. Despite the moderate collinearity, both variables remained statistically significant, supporting the stability of the estimates.

A sensitivity analysis was performed by excluding patients with IL-6 levels above the 95th percentile of the entire study population (*n* = 6, all from the ADHF group). After exclusion, multivariable logistic regression adjusting for age, gender, NT-proBNP, and LVEF demonstrated that IL-6 remained independently associated with ADHF (OR = 1.461, 95% CI: 1.053–2.028, *p* = 0.023), confirming the robustness of our primary findings.

### ROC curve analysis

**Figure 3:**
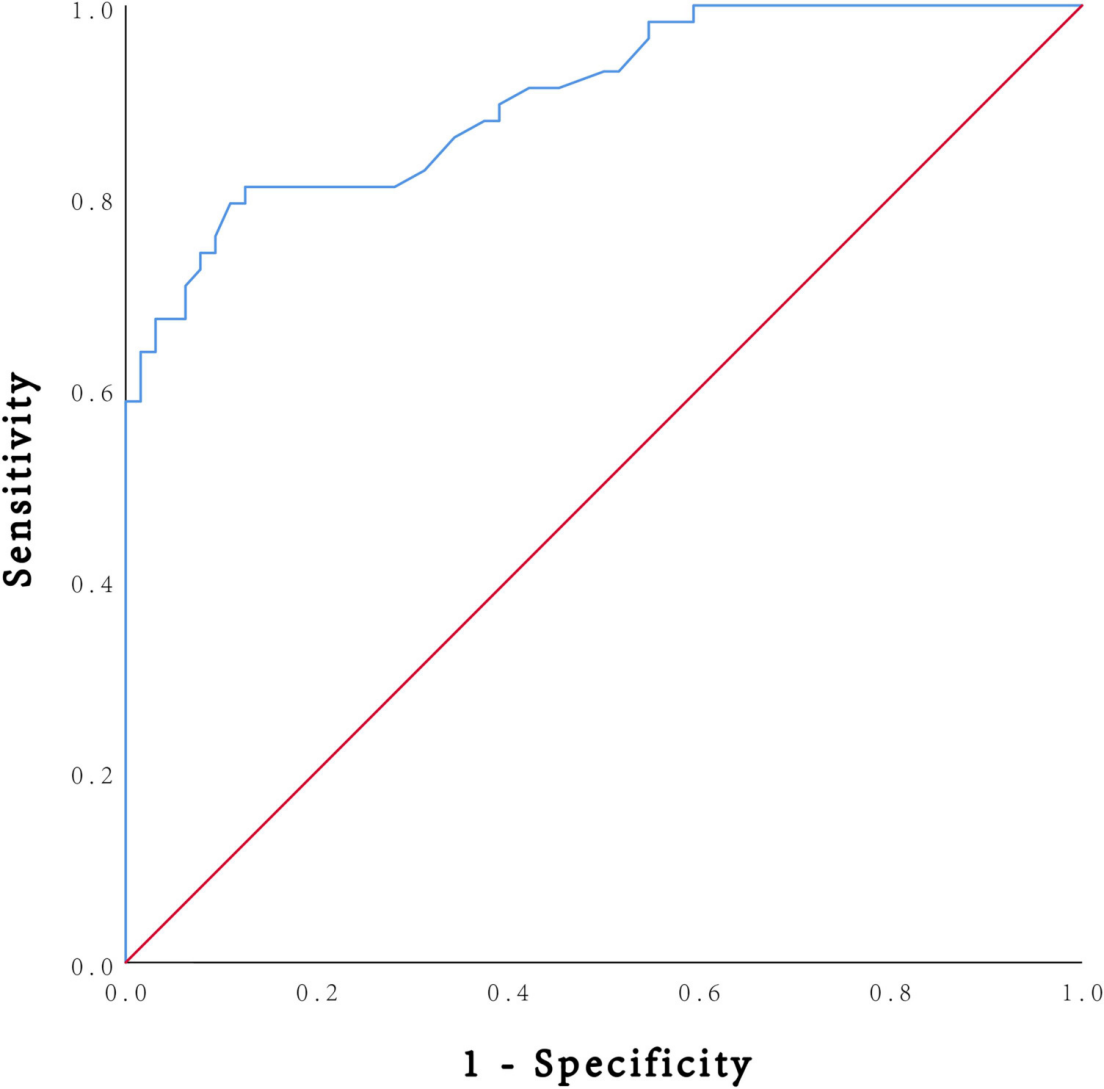
ROC curve of plasma IL-6 for the diagnosis of ADHF. The AUC for IL-6 level was 0.904 (*p* < 0.001), indicating excellent diagnostic discrimination. The optimal cut-off value for IL-6 was determined to be 5.00 pg/mL. At this cut-off value, the sensitivity, specificity and Youden index for ADHF diagnosis were 81.0%, 87.5% and 0.685, respectively. The diagonal line represents the ROC curve for a test with no discriminative ability (AUC = 0.5). ROC, receiver operating characteristic; ADHF, acute decompensated heart failure; AUC, area under the curve.

Based on the above findings suggesting IL-6 as a potential novel diagnostic biomarker for ADHF, we conducted ROC curve analysis to further quantify its diagnostic performance. As shown in Figure 3, the area under the curve (AUC) for IL-6 was 0.904 (*p* < 0.001), indicating excellent diagnostic discrimination. Furthermore, at the optimal cut-off value of 5.00 pg/mL, the sensitivity and specificity for ADHF diagnosis were 81.0% and 87.5%, respectively, corresponding to a Youden index of 0.685.

To formally assess whether IL-6 provides incremental diagnostic value beyond NT-proBNP alone, we compared the performance of two predictive models. Model 1, which included only NT-proBNP, yielded an AUC of 0.987 (95% CI: 0.948–0.999), indicating excellent diagnostic accuracy. Model 2, which included both NT-proBNP and IL-6, yielded a slightly higher AUC of 0.992 (95% CI: 0.956–1.000). The difference in AUC was 0.005, and the DeLong test for correlated ROC curves showed that this difference was not statistically significant (*p* = 0.437).

## Discussion

To investigate the diagnostic value of IL-6 in ADHF, we conducted this retrospective study, which involved patients with either ADHF or CHF. As expected, the plasma levels of IL-6 and NT-proBNP were higher in ADHF patients than in CHF patients. Correlation analyses revealed that IL-6 is positively correlated with NT-proBNP, HR, and LVEDD, and negatively correlated with LVEF. In a logistic regression model, IL-6 remained independently associated with ADHF after adjusting for other variables, such as age, NT-proBNP, and LVEF. Finally, based on the ROC curve, with an optimized IL-6 cut-off value of 5.00 pg/mL, the sensitivity, specificity, and Youden index for the diagnosis of ADHF were 81.0%, 87.5%, and 0.685, respectively. Given its high sensitivity and specificity, IL-6 may have utility as a complementary biomarker to NT-proBNP in discriminating ADHF patients, though this requires confirmation in prospective studies.

HF is the common end-stage manifestation of most heart diseases and may have common clinical presentations, such as dyspnea, fatigue, and edema. ADHF remains associated with a higher mortality rate either in western countries or in China, despite the progress in the treatment and extensive research. As the condition of patients with ADHF can deteriorate rapidly without timely and appropriate treatment, an early and accurate diagnosis and intervention are of particular importance. Although considerable developments have been made in recent years, including the use of BNP or NT-proBNP in ADHF diagnosis, misdiagnosis and missed diagnosis still occur. Hence, developing a diagnostic model that incorporates more biomarkers is essential. Several studies have established the close relationship between HF and inflammatory cytokines, and among the cytokines, IL-6 could be a predictor of advanced HF, as indicated in our study.

Cytokine IL-6 is a pleiotropic inflammatory mediator produced by multiple cell types, including cardiac myocytes, endothelial cells, and immune cells, in response to stress or injury (5). While acute IL-6 may exert cardioprotective effects during immediate myocardial injury, chronic elevation of IL-6 promotes maladaptive changes in the myocardium (9). Mechanistically, persistent IL-6 activation, particularly via trans-signaling, contributes to endothelial dysfunction, myocardial fibrosis, and adverse remodeling (15). The JAK/STAT pathway serves as a key mediator of IL-6 signaling, and its excessive activation contributes to maladaptive inflammation, fibrosis, and HF progression (16, 17).

The correlation patterns observed in our study align with this proposed pathological role of IL-6. Consistent with previous studies, we found that plasma IL-6 was significantly elevated in ADHF patients. Our correlation analyses provide mechanistic insights into IL-6’s role in cardiac dysfunction. The positive correlation between IL-6 and LVEDD suggests that elevated IL-6 may be involved in ventricular remodeling. This is supported by experimental evidence linking IL-6 to cardiomyocyte hypertrophy and extracellular matrix remodeling (18, 19). Rivera et al. also reported correlations between IL-6 and remodeling markers in HF (20). Additionally, the negative correlation between IL-6 and LVEF indicates a potential effect of IL-6 on myocardial contractile function. Inflammatory cytokines, including IL-6, have been shown to suppress cardiomyocyte contractility through multiple mechanisms, including alterations in calcium handling, induction of apoptosis, and *β*-adrenergic receptor desensitization (21). The BIOSTAT-CHF study also confirmed that elevated IL-6 independently predicts adverse outcomes and reduced LVEF (22). Taken together, elevated IL-6 in ADHF patients may not merely be an epiphenomenon but could be mechanistically linked to the disease process by promoting adverse ventricular remodeling and impairing contractility, thereby facilitating the transition from a stable to an acute decompensated state.

The relationship between IL-6 and HF has been further investigated in numerous clinical trials. Matsumoto et al. found that plasma levels of IL-6 and BNP are simultaneously elevated in ADHF and subsequently decrease after treatment (23). Furthermore, a previous study revealed that plasma IL-6 levels are strongly associated with NYHA functional class (24). Thus, plasma IL-6 levels increase with deterioration of heart function. In addition, the increase in plasma IL-6 levels among inpatients with ADHF could predict higher mortality and worse outcomes. Currently, to our knowledge, no studies have reported on the use of plasma IL-6 levels for the diagnosis of ADHF. Our findings suggest that IL-6 could be useful for physicians in clinical practice, specifically in ADHF diagnosis.

Our formal comparison of predictive models showed that adding IL-6 to NT-proBNP provided only a marginal increase in the AUC. This may be attributed to the exceptionally high discriminatory power of NT-proBNP alone in differentiating ADHF from stable CHF in our cohort, leaving limited room for improvement. The primary value of IL-6 in this context may therefore lie not in a major increase in overall discrimination. Rather, it may reflect its independent association with acute decompensation and a distinct inflammatory pathophysiological pathway.

Beyond these primary findings, the robustness of our results was further supported by additional analyses. Within the stable CHF group, IL-6 showed significant correlations with NT-proBNP, NYHA class, and LVEF, indicating that IL-6 correlates with HF severity across the entire disease spectrum. Furthermore, a sensitivity analysis excluding extreme IL-6 values confirmed that the association between IL-6 and ADHF was not driven by outliers. These complementary analyses strengthen the validity of IL-6 as a potential biomarker in HF.

This study has several limitations. First, its retrospective, single-center design may limit the generalizability of the findings, necessitating validation in larger, prospective, multi-center studies. Second, the exclusion of a substantial number of potentially eligible patients due to unavailable IL-6 measurements introduces the potential for selection bias, as the included cohort may not be fully representative of the broader ADHF population. Third, by comparing ADHF specifically to stable CHF, the study design isolates the biomarker's association with acute decompensation but does not address the more common and diagnostically challenging scenario of differentiating ADHF from other acute causes of dyspnea (e.g., pulmonary infections or embolism). Consequently, the reported diagnostic accuracy of IL-6 is likely overestimated relative to its performance in that real-world setting. Fourth, the precise timing of blood sampling relative to symptom onset and initial emergency treatment could not be consistently ascertained, which may influence the interpretation of absolute cytokine levels. Finally, while strict exclusion criteria helped minimize confounding from major conditions known to elevate IL-6, they also limit the applicability of our findings to the more heterogeneous ADHF population encountered in routine clinical practice.

## Conclusions

In conclusion, this retrospective study demonstrated that plasma IL-6 levels were significantly elevated in patients with ADHF and independently associated with the acute decompensated state. The optimal cut-off value of 5.00 pg/mL demonstrated good diagnostic performance, which may serve as a novel diagnostic reference for Chinese patients with ADHF without AMI. These findings provide clinical evidence supporting the role of IL-6 as an inflammation-related biomarker in cardiovascular disease. Furthermore, they suggest the potential value of combining IL-6 with NT-proBNP to improve diagnostic assessment. However, given the observational design and inherent limitations of this study, our findings should be considered preliminary, and future prospective studies are warranted to validate IL-6 as a clinical biomarker for ADHF.

## Data Availability

The original contributions presented in the study are included in the article/Supplementary Material, further inquiries can be directed to the corresponding authors.

## References

[1] Benjamin EJ Virani SS Callaway CW Chamberlain AM Chang AR Cheng S Heart Disease and Stroke Statistics-2018 update: a report from the American Heart Association. Circulation. (2018) 137(12):e67–492. 10.1161/CIR.0000000000000558 29386200

[2] Fonarow GC Stough WG Abraham WT Albert NM Gheorghiade M Greenberg BH Characteristics, treatments, and outcomes of patients with preserved systolic function hospitalized for heart failure: a report from the OPTIMIZE-HF registry. J Am Coll Cardiol. (2007) 50(8):768–77. 10.1016/j.jacc.2007.04.064 17707182

[3] Murphy SP Kakkar R McCarthy CP Januzzi JL Jr. Inflammation in heart failure: JACC state-of-the-art review. J Am Coll Cardiol. (2020) 75(11):1324–40. 10.1016/j.jacc.2020.01.014 32192660

[4] Alogna A Koepp KE Sabbah M Espindola Netto JM Jensen MD Kirkland JL Interleukin-6 in patients with heart failure and preserved ejection fraction. JACC Heart Fail. (2023) 11(11):1549–61. 10.1016/j.jchf.2023.06.031 37565977 PMC10895473

[5] Ridker PM Rane M. Interleukin-6 signaling and anti-interleukin-6 therapeutics in cardiovascular disease. Circ Res. (2021) 128(11):1728–46. 10.1161/CIRCRESAHA.121.319077 33998272

[6] Mehta NN deGoma E Shapiro MD. IL-6 and cardiovascular risk: a narrative review. Curr Atheroscler Rep. (2024) 27(1):12. 10.1007/s11883-024-01259-7 39589436 PMC11599326

[7] Broch K Anstensrud AK Woxholt S Sharma K Tollefsen IM Bendz B Randomized trial of interleukin-6 receptor inhibition in patients with acute ST-segment elevation myocardial infarction. J Am Coll Cardiol. (2021) 77(15):1845–55. 10.1016/j.jacc.2021.02.049 33858620

[8] Docherty KF McDowell K Welsh P Petrie MC Anand I Berg DD Interleukin-6 in heart failure with reduced ejection fraction and the effect of dapagliflozin: an exploratory analysis of the dapagliflozin and prevention of adverse outcomes in heart failure trial. JACC Heart Fail. (2025) 13(7):102393. 10.1016/j.jchf.2024.12.012 40088234

[9] Fontes JA Rose NR Cihakova D. The varying faces of IL-6: from cardiac protection to cardiac failure. Cytokine. (2015) 74(1):62–8. 10.1016/j.cyto.2014.12.024 25649043 PMC4677779

[10] Boulogne M Sadoune M Launay JM Baudet M Cohen-Solal A Logeart D. Inflammation versus mechanical stretch biomarkers over time in acutely decompensated heart failure with reduced ejection fraction. Int J Cardiol. (2017) 226:53–9. 10.1016/j.ijcard.2016.10.038 27788390

[11] Vasan RS Sullivan LM Roubenoff R Dinarello CA Harris T Benjamin EJ Inflammatory markers and risk of heart failure in elderly subjects without prior myocardial infarction: the Framingham Heart study. Circulation. (2003) 107(11):1486–91. 10.1161/01.CIR.0000057810.48709.F6 12654604

[12] Heidenreich PA Bozkurt B Aguilar D Allen LA Byun JJ Colvin MM 2022 AHA/ACC/HFSA guideline for the management of heart failure: a report of the American College of Cardiology/American Heart Association Joint Committee on Clinical Practice Guidelines. Circulation. (2022) 145(18):e895–1032. 10.1161/CIR.0000000000001063 35363499

[13] Authors/Task Force Members, McDonagh TA Metra M Adamo M Gardner RS Baumbach A Böhm M 2023 Focused update of the 2021 ESC guidelines for the diagnosis and treatment of acute and chronic heart failure: developed by the task force for the diagnosis and treatment of acute and chronic heart failure of the European Society of Cardiology (ESC) with the special contribution of the Heart Failure Association (HFA) of the ESC. Eur J Heart Fail. (2024) 26(1):5–17. 10.1002/ejhf.3024 38169072

[14] Thygesen K Alpert JS Jaffe AS Chaitman BR Bax JJ Morrow DA Fourth universal definition of myocardial infarction (2018). J Am Coll Cardiol. (2018) 72(18):2231–64. 10.1016/j.jacc.2018.08.1038 30153967

[15] Preda A Tirandi A Leo G Di Vece D Vilahur G Bonaventura A IL-6 in the spotlight: from cardiovascular pathophysiology to therapy. Eur J Clin Invest. (2026) 56(1):e70161. 10.1111/eci.70161 41340186

[16] Terrell AM Crisostomo PR Wairiuko GM Wang M Morrell ED Meldrum DR. Jak/STAT/SOCS signaling circuits and associated cytokine-mediated inflammation and hypertrophy in the heart. Shock. (2006) 26(3):226–34. 10.1097/01.shk.0000226341.32786.b9 16912647

[17] Yang Q Ji H Modarresi Chahardehi A. JAK/STAT pathway in myocardial infarction: crossroads of immune signaling and cardiac remodeling. Mol Immunol. (2025) 186:206–17. 10.1016/j.molimm.2025.08.018 40882407

[18] Melendez GC McLarty JL Levick SP Du Y Janicki JS Brower GL. Interleukin 6 mediates myocardial fibrosis, concentric hypertrophy, and diastolic dysfunction in rats. Hypertension. (2010) 56(2):225–31. 10.1161/HYPERTENSIONAHA.109.148635 20606113 PMC2921860

[19] Sanders E Kaur K Wagner N Emig R Aronovitz M Bayer AL Endothelial cell stimulator of interferon genes regulates IL-6 production and is required for pathologic cardiac hypertrophy and contractile dysfunction in experimental heart failure. Am J Pathol. (2025) 195(10):1788–807. 10.1016/j.ajpath.2025.06.004 40639717 PMC12597551

[20] Rivera M Talens-Visconti R Jordan A Sirera R Sevilla B Climent V Myocardial remodeling and immunologic activation in patients with heart failure. Rev Esp Cardiol. (2006) 59(9):911–8. 10.1157/13092799 17020704

[21] Hanna A Frangogiannis NG. Inflammatory cytokines and chemokines as therapeutic targets in heart failure. Cardiovasc Drugs Ther. (2020) 34(6):849–63. 10.1007/s10557-020-07071-0 32902739 PMC7479403

[22] Markousis-Mavrogenis G Tromp J Ouwerkerk W Devalaraja M Anker SD Cleland JG The clinical significance of interleukin-6 in heart failure: results from the BIOSTAT-CHF study. Eur J Heart Fail. (2019) 21(8):965–73. 10.1002/ejhf.1482 31087601

[23] Matsumoto M Tsujino T Lee-Kawabata M Naito Y Sakoda T Ohyanagi M Serum interleukin-6 and C-reactive protein are markedly elevated in acute decompensated heart failure patients with left ventricular systolic dysfunction. Cytokine. (2010) 49(3):264–8. 10.1016/j.cyto.2009.11.006 20005739

[24] Torre-Amione G Kapadia S Benedict C Oral H Young JB Mann DL. Proinflammatory cytokine levels in patients with depressed left ventricular ejection fraction: a report from the Studies of Left Ventricular Dysfunction (SOLVD). J Am Coll Cardiol. (1996) 27(5):1201–6. 10.1016/0735-1097(95)00589-7 8609343

